# CHALLENGES AND OPPORTUNITIES IN RECRUITING DIVERSE OLDER ADULTS WHO ARE FRAIL FROM THE MAPS-B STUDY

**DOI:** 10.1093/geroni/igad104.2337

**Published:** 2023-12-21

**Authors:** Suleman Tariq, Alexa Kouroukis, Courtney Kennedy, Jonathan Adachi, Carolyn Leckie, Alexandra Papaioannou, Isabel Rodrigues

**Affiliations:** McMaster University, Hamilton, Ontario, Canada; McMaster University, Hamilton, Ontario, Canada; McMaster University, Hamilton, Ontario, Canada; McMaster University, Hamilton, Ontario, Canada; St. Joseph’s Healthcare Hamilton, Hamilton, Ontario, Canada; McMaster University, Hamilton, Ontario, Canada; McMaster University, Hamilton, Ontario, Canada

## Abstract

Including diverse individuals at the research and participant levels are essential to improve the effectiveness of real-world interventions; however, there are challenges when including such individuals. Our study purpose was to report the challenges of recruiting diverse older adults for the Mapping Sedentary Behaviour study. Our methods were guided by Step 1 (“Establish Partnerships”) in the Knowledge-to-Action-Ethics Framework. We assembled a diverse team of eleven researchers, clinicians, and patient partners. To recruit a broad group of participants, we partnered with City Housing Hamilton, which provides subsidized housing for older adults. We met with the organization’s partnership development advisor who organized two recruitment orientations; 80 potential participants and returning attendees were present for both sessions. The organization provided coffee and donuts. Most attendees were from visible minorities and had visible disabilities (i.e., used a walker or cane). To build rapport, we met with attendees in groups of 5 to 6 to introduce the research team and explain the study. We recruited 13 participants (seven female, one transgender man; Morley FRAIL score≥3). Before their scheduled study visit, twelve participants dropped out citing medical mistrust (i.e., fearing unintentional medical tracking). The last participant dropped out after the initial study visit due to their family’s skepticism in research. Additionally, some individuals may have enrolled for financial incentives as they were interested in receiving immediate monetary compensation. We faced challenges when recruiting frail older adults from diverse backgrounds. Future studies should focus on developing methods to target medical mistrust with older adults and their families.

